# Impact of belly board immobilization devices and body mass factor on setup displacement using daily cone‐beam CT in rectal cancer radiotherapy

**DOI:** 10.1002/acm2.14573

**Published:** 2024-11-29

**Authors:** Junjie Ruan, Xiaotong Huang, Tong Wang, Xiuying Mai, Chuyan Lin, Fanghua Li, Yunfeng Li, Feng Chi, Bin Li

**Affiliations:** ^1^ State Key Laboratory of Oncology in South China Guangdong Provincial Clinical Research Center for Cancer Sun Yat‐sen University Cancer Center Guangzhou P. R. China

**Keywords:** belly board immobilization, body mass factor, cone‐beam CT, rectal cancer radiotherapy, setup displacement

## Abstract

**Objective:**

The objective of this study is to evaluate the impact of different belly board and daily changes in patient's body‐mass factor (BMF) on setup displacement in radiotherapy for rectal cancer.

**Methods:**

Twenty‐five patients were immobilized using the thermoplastic mask with belly board (TM‐BB), and 30 used the vacuum bag cushion with belly board (VBC‐BB), performing daily cone‐beam computed tomography (CBCT) scans 625 times and 750 times, respectively. Daily pretreatment CBCT scans were registered to the planned CT images for BMF change determination and setup displacement measurement. Independent t‐tests compared setup displacement between the two groups in left‐right (LR), superior‐inferior (SI), and anterior‐posterior (AP) directions, as well as the BMF changes. The impact of daily BMF changes on setup displacement was evaluated using multivariate logistic regression and 10‐fold cross‐validation.

**Results:**

The setup displacement for TM‐BB in the LR, SI, and AP directions were 0.31 ± 0.25, 0.58 ± 0.40, and 0.19 ± 0.18 cm, respectively, while VBC‐BB showed 0.19 ± 0.15, 0.26 ± 0.22, and 0.36 ± 0.29 cm in the corresponding directions, respectively. Margins of planning target volume (PTV) for TM‐BB were 8, 10, and 6 mm in LR, SI, and AP directions, while VBC‐BB showed margins of 5,7, and 8 mm, respectively. The daily BMF changes for both groups were ranked in descending order as follows: sacral rotation angle (RS), hip lateral diameter (HLD), and hip anterior‐posterior diameter (HAPD). HAPD was the main factor affecting setup displacement in both the AP and SI directions in TM‐BB, while RS was the primary factor for setup displacement in the AP direction in VBC‐BB.

**Conclusion:**

Compared with TM‐BB, VBC‐BB had a larger AP displacement but smaller in LR and SI displacement. Daily changes in BMF have distinct effects on setup displacement in different immobilization devices. Image‐guided radiation therapy (IGRT) is highly recommended and BMF changes should be given consideration during radiotherapy.

## 1 INTRODUCTION

Colorectal cancer (CRC) is the third most common malignancy, with the third‐highest incidence rate and second‐highest mortality rate.^1^, ^2^ Radical surgery is the preferred treatment approach for early‐stage CRC. However, nearly 50% of CRC patients with initial primary diagnosis have progressed to locally advanced stage, which cannot receive radical surgery.^3^, ^4^ Radiotherapy is a significant treatment approach for locally advanced stage CRC.^5^, ^6^ Due to tumor volume monitoring and organ motion in the real‐time by using cone‐beam computed tomography (CBCT), image‐guided radiation therapy (IGRT) has been recommended for the treatment of CRC. IGRT not only ensures that the radiation dose is accurately delivered to the tumor volume while minimizing radiation exposure to organs at risk^7^, ^8^ but also allows the measurement of setup displacement during treatment,^9^, ^10^ providing reliable quantitative basis for evaluating immobilization devices. Selecting different immobilization devices for CRC patients can induce the setup displacement and radiotherapy toxicity response distinctively.^11^, ^12^, ^13^

The belly board (BB) is more effective in distancing abdominal organs from the planning target volume, which reduces radiation exposure to the small intestine and alleviate radiation enteritis.^14^ However, the BB has been found to cause discomfort to the patient and to reduce the setup reproducibility compared with patients who were immobilized using the vacuum bag cushion (VBC).^15^, ^16^ Previous studies have investigated the thermoplastic mask with belly board (TM‐BB), which has been used in the treatment of pelvic tumors aimed at improving setup reproducibility.^17^, ^18^, ^19^ However, TM‐BB may cause pressure for patients and may loosen during treatment.^20^, ^21^ Therefore, we have developed an innovative immobilization device that combines VBC and BB (VBC‐BB) to improve the setup repeatability and comfort of the patient. Due to the extended treatment cycle, weight fluctuations, and skin laxity during radiotherapy for CRC,^22^, ^23^, ^24^, ^25^, ^26^, ^27^, ^28^ the patient's body‐mass factor (BMF) changes can be an important factor affecting setup repeatability. Previous studies have investigated the impact of pre‐treatment BMF on gynecological and head and neck tumors.^29^, ^30^, ^31^ However, few studies have investigated impact of intra‐fraction BMF changes on radiotherapy for CRC.

This study aims to compare the TM‐BB and VBC‐BB immobilization devices in rectal cancer radiotherapy setup displacement using daily CBCT. Additionally, it investigates the potential impact of daily BMF changes on the setup displacement for both devices. The objective is to assess the impact of different immobilization devices and daily BMF changes on setup displacement in patients with CRC.

## 2 MATERIALS AND METHODS

### 2.1 Analysis of baseline patient characteristics

This retrospective analysis examined patients diagnosed with CRC who underwent radiotherapy at our institution from March 2021 to December 2022. The TM‐BB group consisted of 25 patients, and the VBC‐BB group included 30 patients. No statistically significant differences (*p* > 0.05) were found between the two groups regarding gender or other relevant factors. All patients followed a bladder and bowed preparation protocol that required an empty rectum and a full bladder, starting one week before the CT simulation and continuing throughout the treatment.

### 2.2 Fabrication step of immobilization device

#### 2.2.1 TM‐BB immobilization device

**FIGURE 1:**
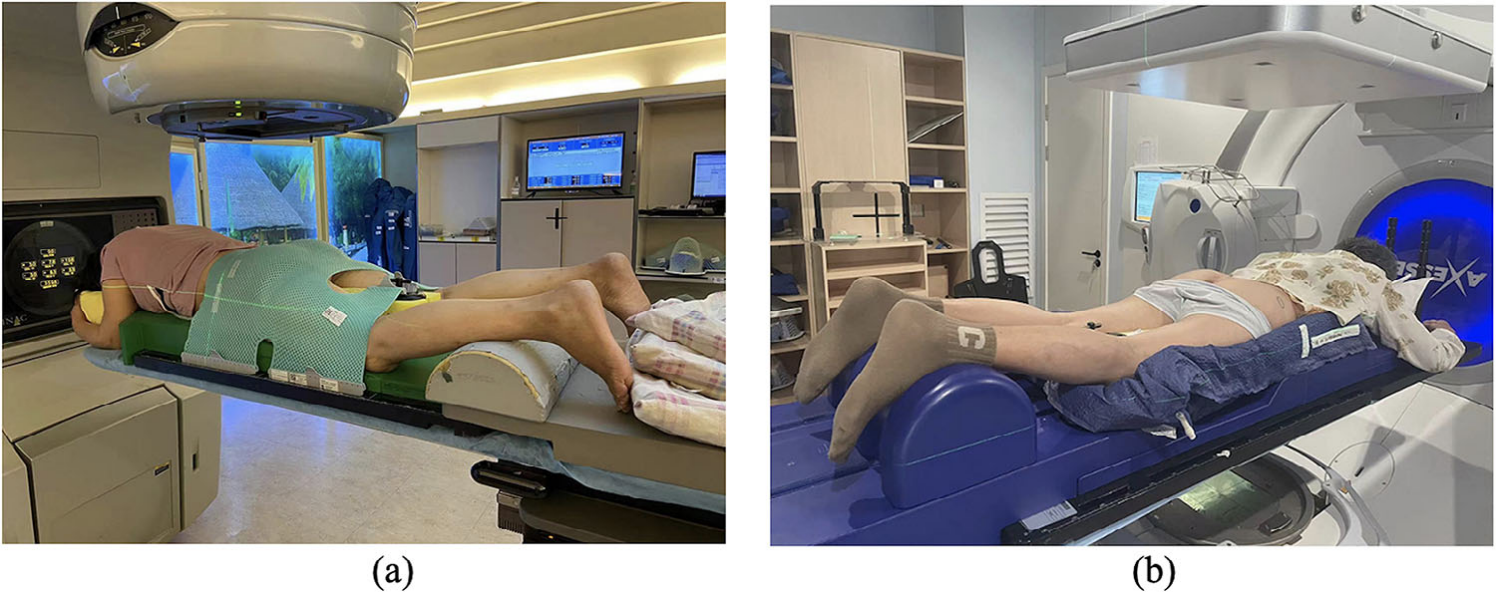
Immobilization devices: (a) thermoplastic mask with belly board; (b) vacuum bag cushion with belly board.

The patients were then positioned prone on the BB (Orfit industries, Wijnegem, Belgium) after the appropriate size of the abdominal hole and crotch block was selected based on the patient's abdominal circumference and height. The legs of the patients were immobilized using the footrest. Finally, the TM was softened with hot water and applied to the patient's body surface, as shown in Figure 1a. The process of cooling and shaping the TM took approximately 15 min.

#### 2.2.2 VBC‐BB immobilization device

The size of the abdominal hole, the crotch block, and the VBC was selected according to the characteristics of each patient. Patients were then positioned prone on the VBC after placing the VBC on the BB (Tengfeiyu industries, Shenzhen, China). The VBC was then shaped to match the patient's body contour using a vacuum pump. The patient's hands were immobilized with a support bar while the legs were immobilized with footrests, as shown in Figure 1b.

### 2.3 Preparation for bladder filling and rectal emptying

Patients received dietary modifications one week before the procedure and underwent daily training for urine retention to develop a habit of urine retention. Before the initiation of position fixation and simulation CT for radiation therapy (CT‐Sim), patients were instructed to empty their bladders one hour in advance. To ensure proper bowel preparation, they were advised to use a glycerol enema and dimethicone to expel rectal gas and fecal matter. Afterward, patients were instructed to drink 1000 mL of water to facilitate adequate bladder filling. Positioning fixation and CT‐Sim were then initiated only after patients reported urinary urgency and underwent ultrasonic cystometry (BladderScan, BVI 9400) to confirm bladder volume was between 300 and 500 mL. The patient's bladder capacity and retention time were documented.

### 2.4 CT simulation (CT‐Sim)

The SIEMENS Somatom machine was used for CT‐SIM with the following parameters: slice thickness and spacing of 2.5 mm each, tube voltage set at 120 kV, tube current between 50 and 440 mAs, and 1.375:1 mm pitch. The placement of radiopaque fiducial markers differed significantly between the two groups. The VBC‐BB group used a laser to mark crosses at 90°, 0°, and 270° on the patient's body surface and placed the radiopaque fiducial markers accordingly. In the TM‐BB group, the laser was used to mark crosses at 90°, 0°, and 270° on the patient's body surface. The patient's body surface was then covered with the TM. The cross lines were marked at corresponding locations on the TM and radiopaque fiducial markers were placed.

### 2.5 Target volume definition and treatment planning

Gross tumor volume (GTV), Clinical target volume (CTV), planning target volume (PTV), and organs at risk (OAR) were contoured by an experienced oncology radiation specialist according to RTOG standards. The physicists used the volumetric modulated arc therapy (VMAT) technique on the Eclipse planning system to create treatment plans. The treatment plan was for a total dose of 50 Gy delivered in 25 fractions using the Varian Halcyon linear accelerator with 6 MV photons. The oncology radiation specialist then reviewed and approved the treatment plans.

### 2.6 Daily CBCT verification and setup displacement measurement

The cross marks on the patient's surface were aligned with the laser cross lines. CBCT scanning was performed before each treatment fraction with parameters of 125 kV and 1080 mAs, and a reconstructed slice thickness of 2 mm. First, the CBCT images were automatically registered with grayscale algorithm to the planned CT images. Manual registration was then performed with a focus on the clinical target volume (CTV) to obtain the setup displacement values in LR (left‐right), SI (superior‐inferior), and AP (anterior‐posterior) directions. During this process, the window level was adjusted to a range of 35 to 40 Hounsfield units (HU) and the window width was set to a range of 250–350 HU. Finally, the setup displacement was then corrected by the linear accelerator system and recorded for each treatment fraction.

### 2.7 Measurement of BMF changes

**FIGURE 2:**
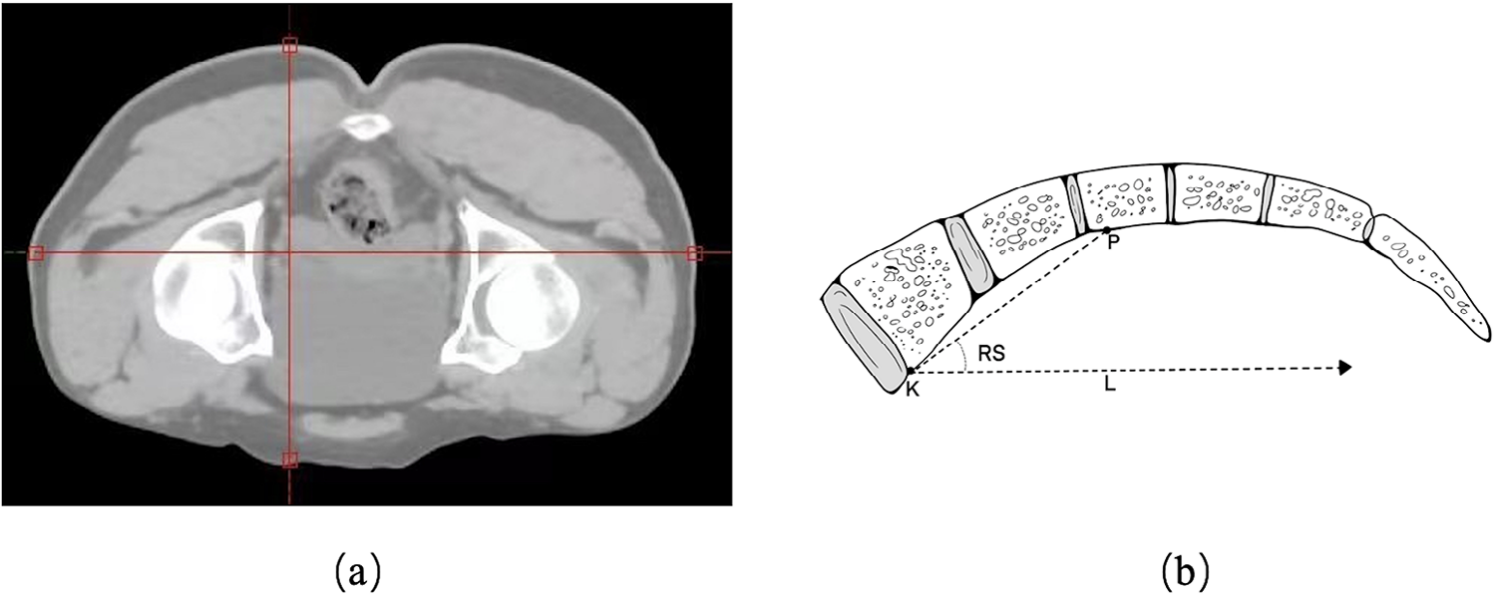
Measurement of body mass factor in the planning CT image. (a) Measurement of hip anterior‐posterior diameter and hip lateral diameter; (b) measurement of rotation of sacral angle.

The hip joint plane was defined as the top of the femoral head. The hip anterior‐posterior diameter (HAPD) and the hip lateral diameter (HLD) were represented as the maximal diameters of the hip joint plane in the AP and LR directions, respectively, as shown in Figure 2a. The point k was drawn in the S1 vertebral margin. A horizontal line L was drawn along point K. A line of 6 cm in length was then drawn at point K, which rotates counterclockwise around point K until it intersects the sacral bone margin. The intersection point was delineated as point p. The line segment KP was formed by connecting points K and P, and the sacral rotation angle (RS) was delineated as the angle between the line KP and the line L,^32^ as shown in Figure 2b.

The BMF changes were calculated by comparing measurements from the planning CT and CBCT images. Equations ((1), (2), (3)) for calculating the absolute change in HAPD (ACHAPD), HLD (ACHLD), and RS (ACRS) calculations are provided below:

(1) $$ACHPAD=\left|HAPD\left(CBCT\right)-HAPD\left(CT\right)\right|\;,$$

(2) $$ACHLD=\left|HLD\left(CBCT\right)-HLD\left(CT\right)\right|\;,$$

(3) $$ACRS=\left|RS\left(CBCT\right)-RS\left(CT\right)\right|\;,$$

### 2.8 Statistical analysis and tenfold cross‐validation

Continuous variables were presented as mean ± standard deviation (mean ± SD) and analyzed using independent samples *t*‐tests. For the continuous variables that do not meet the normality assumption, we employed the Mann‐Whitney *U* test for analysis. Categorical variables were analyzed using Chi‐square tests and presented as frequencies (*n*). Statistical significance was determined at *p*‐value < 0.05. PTV margins were calculated using Van Herk's formula,^33^ margins of PTV (MPTV)=2.5Σ+0.7σ, where systematic (Σ) and random (σ) errors were considered. In Van Herk's formula, systematic (Σ) errors are the SD of the means per patient and random (𝜎) errors are group means of the SD of the random error. The group mean is determined by the root mean square of the SDs of all patients. The effect of daily BMF changes on setup displacement, which was divided into high‐risk and low‐risk groups based on their mean, was investigated using multiple logistic regression.

The setup displacement data were divided into training and validation sets through 10‐fold cross‐validation. In the training sets, the optimal BMF cutoff was calculated by receiver operating characteristic (ROC) curve when BMF was statistically significant in the multiple logistic regression. The setup displacement of the validation sets was classified into high‐risk and low‐risk groups based on the optimal BMF cutoff determined from the training sets and compared. All statistical analyses were performed using IBM SPSS Statistics version 21.0 (IBM, Armonk, New York, USA).

## 3 RESULTS

### 3.1 Comparison of setup displacement and PTV margins between groups

**FIGURE 3:**
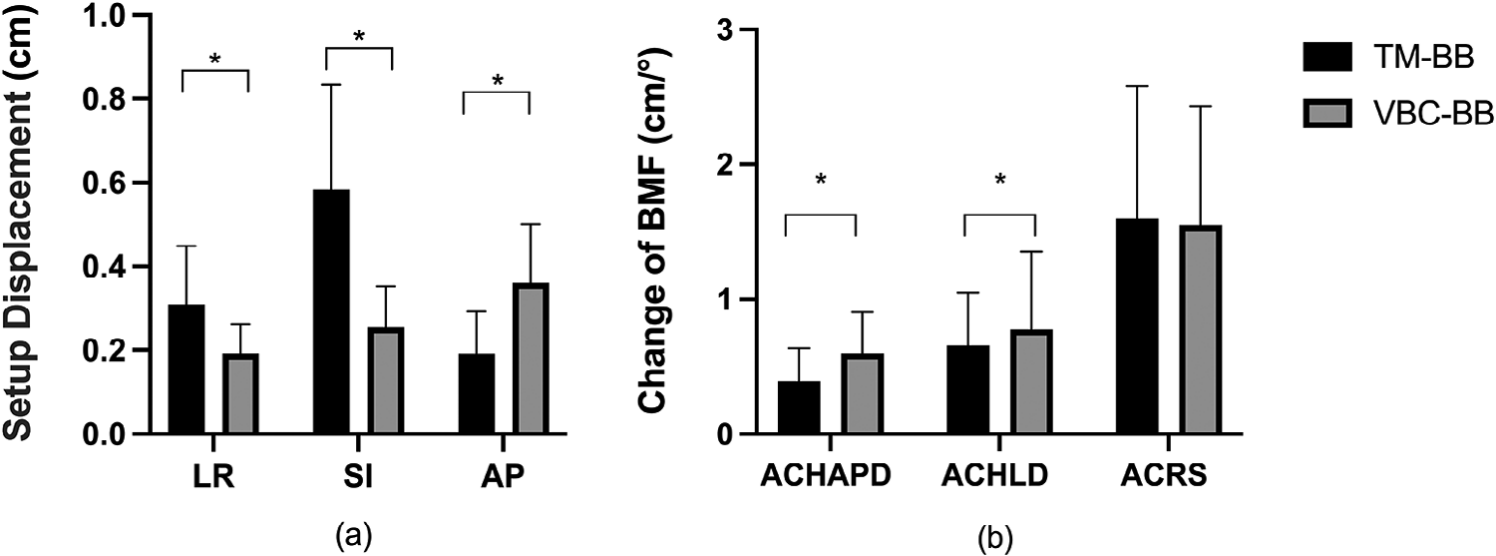
Comparison of (a) setup displacement and (b) body mass factor changes between two immobilization devices.

Setup displacement in the TM‐BB group in the LR, SI, and AP directions were 0.31 ± 0.25, 0.58 ± 0.40, and 0.19 ± 0.18 cm, while those in the VBC‐BB group were 0.19 ± 0.15, 0.26 ± 0.22, and 0.36 ± 0.29 cm, respectively, as shown in Figure 3a. PTV margins in the LR, SI, and AP directions were 8, 10, and 6 mm in the TM‐BB group and 5, 7, and 8 mm in the VBC‐BB group, respectively, as shown in Table 1. Compared with the TM‐BB group, the VBC‐BB group had decreased setup displacement and PTV margins in the LR and SI directions (*p* < 0.01), but was increasing in the AP directions (*p* < 0.01).

**TABLE 1:** Comparison of PTV margins for two immobilization devices.

Direction	Group	SE	RE	MPTV
LR	TM‐BB	0.240	0.277	0.795
	VBC‐BB	0.164	0.187	0.541
SI	TM‐BB	0.279	0.365	0.955
	VBC‐BB	0.218	0.257	0.725
AP	TM‐BB	0.172	0.199	0.569
	VBC‐BB	0.258	0.275	0.836

Abbreviations: AP, anterior‐posterior; LR, left‐right; MPTV, margin of planning target volume; RE, random error; SE, systematic error; SI, superior‐inferior.

### 3.2 Comparison of BMF changes between two immobilization devices

The ACHAPD values were 0.39 ± 0.35 cm in the TM‐BB group and 0.60 ± 0.48 cm in the VBC‐BB group, while the ACHLD values were 0.66 ± 0.55 cm and 0.78 ± 0.72 cm. Similarly, the ACRS were 1.6 ± 1.48° and 1.55 ± 1.27° in the two groups, respectively, as shown in Figure 3b. The TM‐BB group showed a significantly smaller change in both HAPD and HLD (*p* < 0.05) compared with the VBC‐BB group. Additionally, no statistically significant difference was observed in ACRS between the two groups.

### 3.3 Multiple logistic regression of setup displacement factors

**FIGURE 4:**
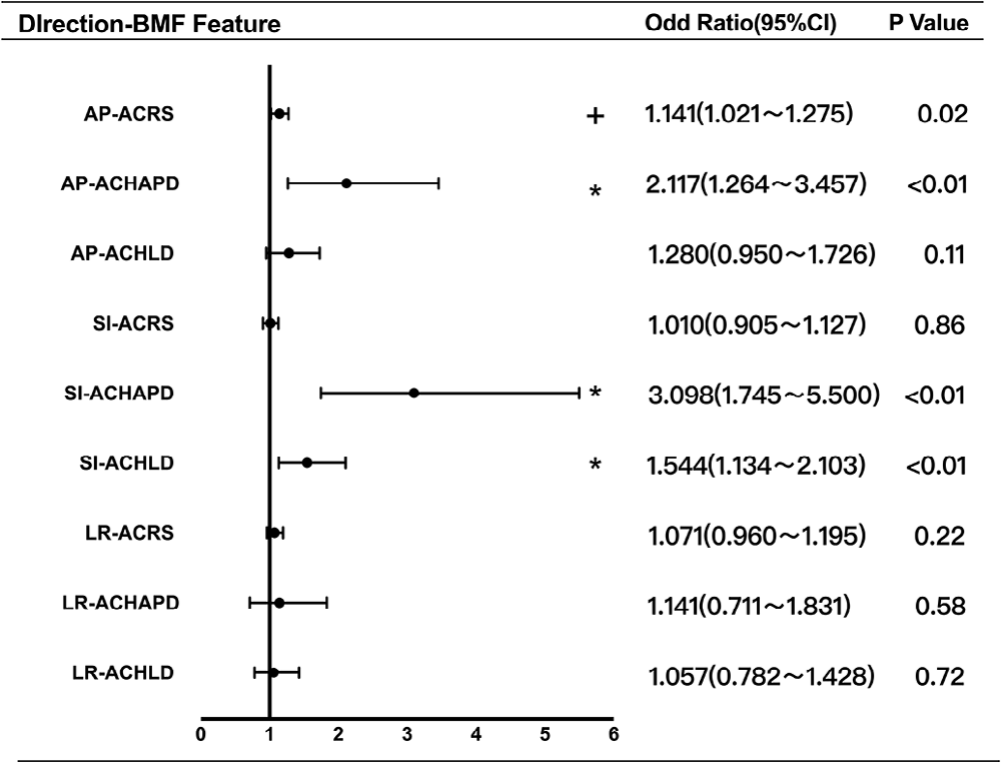
Multivariate analysis of setup displacement for thermoplastic mask with belly board.

In the TM‐BB group, increases in ACRS [odds ratio (OR): 1.141, 95% confidence interval (95% CI): 1.021–1.275, *p* < 0.05] and ACHAPD (OR:2.117, 95% CI: 1.264–3.457, *p* < 0.01) are associated with an increased risk of setup displacement in the AP direction. Increased ACHAPD (OR: 3.098, 95% CI: 1.745–5.500, *p* < 0.01) and ACHLD (OR: 1.544, 95% CI: 1.134–2.103, *p* < 0.01) are associated with an elevated risk of setup displacement in the SI direction. However, the BMF does not significantly impact the risk of setup displacement in the LR direction (*p* > 0.05), as shown in Figure 4.

**FIGURE 5:**
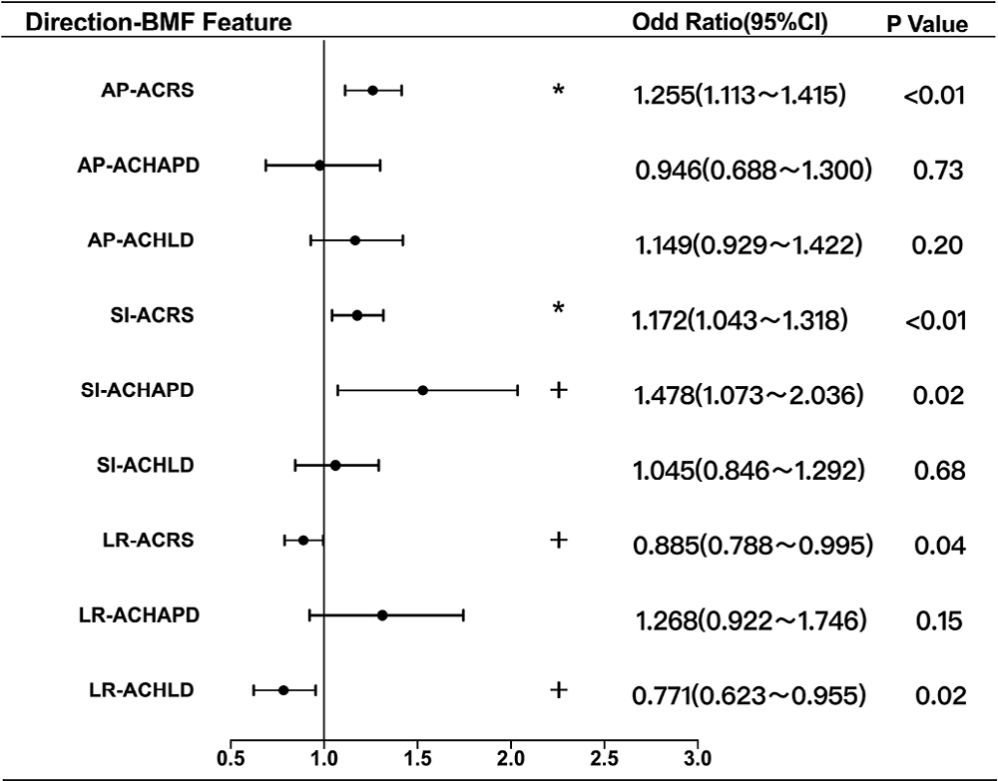
Multivariate analysis of setup displacement for vacuum bag cushion with belly board.

In the VBC‐BB group, increased ACRS (OR: 1.255, 95% CI: 1.113–1.415, *p* < 0.01) is associated with an increase in the risk of setup displacement in the AP direction. Additionally, increases in ACRS (OR: 1.172, 95% CI: 1.043–1.318, *p* < 0.01) and ACHAPD (OR: 1.478, 95% CI: 1.073–2.036, *p* < 0.05) are associated with an increased risk of setup displacement in the SI direction. Conversely, higher ACRS (OR: 0.885, 95% CI: 0.788–0.995, *p* < 0.05) and ACHLD (OR: 0.771, 95% CI: 0.623–0.955, *p* < 0.05) are associated with a reduced risk of setup displacement in the LR direction, as shown in Figure 5.

### 3.4 Cross‐validation

In the TM‐BB group, ACHAPD and ACRS are significantly distinct between high and low‐risk groups for setup displacement in the AP direction (*p* < 0.05). In the SI direction, ACHAPD and ACHLD also show significant differences (*p* < 0.05). Moreover, ACHAPD is effective at differentiating between high and low‐risk groups in both AP and SI directions compared with ACRS and ACHLD (*p* < 0.01), as shown in Table 2.

**TABLE 2:** Risk stratification of setup displacement by body mass factor of thermoplastic mask with belly board.

Direction	BMF	Group	Setup displacement (cm)	*p*	MPTV (cm)	Cutoff (^o^/cm)
AP	ACRS	High Risk	0.221 ± 0.220	<0.05	0.33	2.04
		Low Risk	0.179 ± 0.158		0.27	
	ACHAPD	High Risk	0.302 ± 0.196	<0.01	0.65	0.76
		Low Risk	0.180 ± 0.174		0.25	
SI	ACHAPD	High Risk	0.671 ± 0.421	<0.01	0.89	0.59
		Low Risk	0.555 ± 0.388		0.40	
	ACHLD	High Risk	0.644 ± 0.461	<0.05	0.68	1.00
		Low Risk	0.563 ± 0.374		0.51	

Abbreviations: ACHAPD, absolute change of hip anterior posterior diameter; ACHLD, absolute change of hip lateral diameter; ACRS, absolute change in rotation of sacral angle; AP, anterior posterior; MPTV, margin of planning target volume; SI, superior inferior.

In the VBC‐BB group, ACRS is significantly distinct differentiates between the high‐risk and low‐risk groups in the AP direction (*p* < 0.01). However, neither ACRS nor ACHAPD significantly differentiates the risk in the SI direction (*p* > 0.05). While both ACRS and ACHLD significantly differentiates the risk groups in the LR direction (*p* < 0.05), there is no significant difference in PTV margins between the high‐risk and low‐risk groups, as shown in Table 3.

**TABLE 3:** Risk stratification of setup displacement by body mass factor of vacuum bag cushion with belly board.

Direction	BMF	Group	Setup displacement (cm)	*p*	MPTV (cm)	Cutoff (^o^/cm)
AP	ACRS	High Risk	0.403 ± 0.279	<0.01	0.51	1.15
		Low Risk	0.313 ± 0.208		0.39	
SI	ACRS	High Risk	0.259 ± 0.227	0.68	0.40	1.88
		Low Risk	0.253 ± 0.213		0.39	
	ACHAPD	High Risk	0.271 ± 0.224	0.20	0.35	0.78
		Low Risk	0.249 ± 0.217		0.33	
LR	ACRS	High Risk	0.206 ± 0.164	<0.05	0.26	0.98
		Low Risk	0.183 ± 0.143		0.25	
	ACHLD	High Risk	0.200 ± 0.156	<0.01	0.27	1.04
		Low Risk	0.166 ± 0.138		0.27	

Abbreviations: ACHAPD, absolute change of hip anterior posterior diameter; ACHLD, absolute change of hip lateral diameter; ACRS, absolute change in rotation of sacral angle; AP, anterior‐posterior;LR, left‐right; MPTV, margin of planning target volume; SI, superior‐inferior.

## 4 DISCUSSION

This study aimed to investigate the impact of different BB and daily BMF changes on patient setup displacement during rectal cancer radiotherapy. In our study, we found that VBC‐BB showed increased setup displacement in the AP direction but decreased displacement in LR and SI directions, compared with the TM‐BB. There were few studies to compare the immobilization devices of VBC‐BB with the TM‐BB, but some studies^17^, ^20^ investigated the setup displacement of VBC or TM individually and found that setup displacement had large variations in the SI direction with TM, due to the loosening of the TM and weight loss during treatment. Ikeda et al.^21^ found that using TM had small displacements in the AP direction, and this may due to the fact that TM could effectively reduce respiratory motion. Pelvic muscle tension may also affect the setup accuracy especially in the AP direction,^28^ this would inevitably impact the reproducibility of marker position when using VBC. Inui et al.^19^ found that air leakage during treatment could cause the settling of the VBC, and the mean shifts along the AP direction were approximately 1 mm greater in patients immobilized on the vacuum fixation compared with the hydraulic fixation after the twentieth treatment.

The optimal CTV‐PTV margin can be determined by measuring patient setup displacement, to ensure adequate dose to the CTV.^33^ Different immobilization devices may induce distinct PTV margin for rectal cancer radiotherapy. For instance, Reham et al.^22^ recommended PTV margins were 10, 9, and 11 mm using BB or Vac‐Lok, while Bansal et al.^17^ found that the PTV margins were 5, 18, and 7 mm using TM in the LR, SI, and AP directions, respectively. Our study found that the induced PTV margins of TM‐BB group were 8, 10, and 6 mm, while VBC‐BB group show PTV margins of 5, 7, and 8 mm in the LR, SI, and AP direction. Compared with Bansal's study,^17^ our study found a smaller PTV margin in the SI direction for the TM‐BB group. This difference may be due to two main factors: firstly, the smaller sample size in Bansal's study, which included only seven patients and 135 sets of CBCT data. Second, TM‐BB device allows for the selection of an appropriately sized abdominal hole based on the patient's abdominal circumference. Compared with the TM, the VBC fixes the patient in a “U” shape from the bottom up, whereas the TM fixes the patient from the top down. This design may effectively restrict displacement in the SI direction when the patient's abdominal circumference decreases, resulting in a smaller PTV margin in the SI direction for the VBC‐BB group. However, the positioning markers for the VBC‐BB group are placed directly on the patient's skin, which may lead to an increased PTV margin in the AP direction for the VBC‐BB group compared with the TM‐BB group, especially when there are significant changes in the patient's RS, air leaks in the vacuum bag cushion, or weight loss. In order to achieve adequate CTV coverage, the RTOG Contouring Atlas recommends that PTV margin was between 7.0 and 10.0 mm.^34^ This margin is assumed to account for all setup uncertainties, which incorporate the various techniques of treatment delivery with or without a bowel displacement device. In our study, the PTV margins for both immobilization devices were within the range recommended by RTOG. In addition, IGRT is highly recommended for rectal cancer patients. IGRT not only can reduce impact of setup displacement on PTV margin, but also can monitor rectal motion, bladder filling, and other internal organ movements.^23^, ^24^

BMF changes during treatment are a critical factor influencing setup displacement and show differences with different immobilization devices. In the TM‐BB group, changes in HAPD have a significant effect on setup displacement compared with changes in HLD and RS. This may be due to changes in body weight and pelvic muscle tension affecting HAPD changes. In addition, RS changes should be a concern in VBC‐BB group. Kasabasić et al.^32^ found that RS changes of pelvic tumors in prone position using BB was up to 14 degrees during treatment, resulting in setup displacement of up to 15 mm. If significant changes occur in BMF, the target volume may have moved beyond the designated PTV margin. While pre‐treatment pelvic training can help mitigating changes in RS during treatment,^35^ BMF monitoring remains a challenge. Moore et al.^36^ used an interferometer to capture surface motion and generate dynamic sequences of body height maps. Their findings revealed that surface changes remained within 3 mm during irradiation. Additionally, surface motion was observed primarily along the back. This surface motion affects the movement of the spine and bladder across the 95% isodose contour. Optical surface monitoring systems (OSMS), which have rapid non‐invasive and non‐ionizing radiation characteristics, are increasingly being used in clinical practice.^37^ As patient surface is not blocked by VBC, it is feasible to use OSMS to assist patient setup when using VBC‐BB.

This study was subject to several limitations. First, this study was limited to provide a comprehensive investigation of pre‐treatment BMF on setup displacement because of the small sample size. Second, this study only focused on BMF changes of the hip joint plane and RS changes, due to that the other BMF cannot be extracted from the limited scanning range of the CBCT in the SI direction. Finally, our study did not consider the impact of body weight and abdominal circumference on setup displacement. As weight loss and the BMI categories of patients are significant factors affecting the uncertainty of setup and BMF,^29^, ^30^, ^38^, ^39^ these variables would be considered in the future work.

## 5 CONCLUSION

The VBC‐BB group is more effective in limiting setup displacement in the LR and SI directions, while it also shows greater setup displacement in the AP direction, compared with the TM‐BB group. The daily BMF changes during treatment have distinct effects on setup displacement in different immobilization devices groups. IGRT is highly recommended and BMF changes should be given consideration during radiotherapy.

## AUTHOR CONTRIBUTIONS

Conception and design: Bin Li; Administrative support: Bin Li; Provision of study materials or patients: Feng Chi, Tong Wang, Xiuying Mai; Collection and assembly of data: Junjie Ruan, Xiaotong Huang, Chuyan Lin; Data analysis and interpretation: Junjie Ruan, Xiaotong Huang, Fanghua Li, Yunfeng Li; Manuscript writing: All authors; Final approval of manuscript: All authors.

## CONFLICT OF INTEREST STATEMENT

The authors declare no conflicts of interest.

## ETHICS STATEMENT

The study was conducted in accordance with the Declaration of Helsinki. The study was approved by the Ethics Committee of Sun Yat‐sen University Cancer Center (No. B2024‐459‐01) and the requirement for individual consent for this retrospective analysis was waived.
